# Polyhydroxyalkanoate (PHA) Polymer Accumulation and *pha* Gene Expression in Phenazine (phz^-^) and Pyrrolnitrin (prn^-^) Defective Mutants of *Pseudomonas chlororaphis* PA23

**DOI:** 10.3390/polym10111203

**Published:** 2018-10-27

**Authors:** Parveen K. Sharma, Riffat I. Munir, Jocelyn Plouffe, Nidhi Shah, Teresa de Kievit, David B. Levin

**Affiliations:** 1 Department of Biosystems Engineering, University of Manitoba, Winnipeg, MB R3T 5V6, Canada; parveen.sharma@umanitoba.ca (P.K.S.); riffat2munir@yahoo.com (R.I.M.); 2 Department of Microbiology, University of Manitoba, Winnipeg, MB R3T 5V6, Canada; jocplouffe@hotmail.com (J.P.); nidhi888@hotmail.com (N.S.); teresa.dekievit@umanitoba.ca (T.d.K.)

**Keywords:** *Pseudomonas chlororaphis* PA23, Biocontrol, Phenazine, Pyrrolnitrin, *phz*^-^ and *prn*^-^ mutants, Regulatory mutants, *gacS*, *rpoS*, *relA/spoT*, Polyhydroxyalkanoates, *pha* gene expression

## Abstract

*Pseudomonas chlororaphis* PA23 was isolated from the rhizosphere of soybeans and identified as a biocontrol bacterium against *Sclerotinia sclerotiorum*, a fungal plant pathogen*.* This bacterium produces a number of secondary metabolites, including phenazine-1-carboxylic acid, 2-hydroxyphenazine, pyrrolnitrin (PRN), hydrogen cyanide, proteases, lipases and siderophores. It also synthesizes and accumulates polyhydroxyalkanoate (PHA) polymers as carbon and energy storage compounds under nutrient-limited conditions. Pseudomonads like *P. chlororaphis* metabolize glucose via the Entner-Doudoroff and Pentose Phosphate pathways, which provide precursors for phenazine production. Mutants defective in phenazine (PHZ; PA23-63), PRN (PA23-8), or both (PA23-63-1) accumulated higher concentrations of PHAs than the wild-type strain (PA23) when cultured in Ramsay’s Minimal Medium with glucose or octanoic acid as the carbon source. Expression levels of six *pha* genes, *phaC1*, *phaZ*, *phaC2*, *phaD*, *phaF*, and *phaI*, were compared with wild type PA23 by quantitative real time polymerase chain reaction (qPCR). The qPCR studies indicated that there was no change in levels of transcription of the PHA synthase genes *phaC1* and *phaC2* in the *phz^-^* (PA23-63) and *phz^-^* *prn^-^* (PA23-63-1) mutants in glucose medium. There was a significant increase in expression of *phaC2* in octanoate medium. Transcription of *phaD*, *phaF* and *phaI* increased significantly in the *phz^-^* *prn^-^* (PA23-63-1) mutant. Mutations in regulatory genes like *gacS*, *rpoS*, and *relA/spoT*, which affect PHZ and PRN production, also resulted in altered gene expression. The expression of *phaC1*, *phaC2*, *phaF*, and *phaI* genes was down-regulated significantly in *gacS* and *rpoS* mutants. Thus, it appears that PHZ, PRN, and PHA production is regulated by common mechanisms. Higher PHA production in the *phz^-^* (PA23-63), *prn*- (PA23-8), and *phz^-^* *prn^-^* (PA23-63-1) mutants in octanoic medium could be correlated with higher expression of *phaC2*. Further, the greater PHA production observed in the *phz^-^* and *prn^-^* mutants was not due to increased transcription of PHA synthase genes in glucose medium, but due to more accessibility of carbon substrates and reducing power, which were otherwise used for the synthesis of PHZ and PRN.

## 1. Introduction

*Pseudomonas chlororaphis* strain PA23 is a soybean rhizosphere isolate that has been developed as a biocontrol agent to protect canola from stem rot caused by *Sclerotinia sclerotiorum* [1,2]. Strain PA23 produces a number of compounds, including phenazines (PHZ), pyrrolnitrin (PRN), hydrogen cyanide (HCN), proteases, lipases and siderophores that contribute to its biological control potential [3,4]. PHZs are bacterial secondary metabolites that have long been recognized for their broad-spectrum antibiotic activity and ability to antagonize a range of fungal phytopathogens [5,6,7]. Phenazine-1-carboxylic acid (PCA), one of the major PHZs produced by the fluorescent pseudomonads, was commercially named as Shenqinmycin. A 1% Shenqinmycin suspension was registered as a new biopesticide to prevent rice sheath blight, pepper blight and cucumber seedling damping-off [8]. 

Glucose and glycerol favor the production of PHZ in *P. chlororaphis* PCL1391 [9], while mannitol and fructose favor the production of PRN in *P. protegens* strain CHA0 [10]. PHZs are synthesized from glucose via the shikimic acid pathway [11]. A number of mutants defective in PHZ or PRN production, or both, were isolated from *P. chlororaphis* PA23 [12]. These mutations affected *P. chlororaphis* antibiotic production as well as biocontrol activity [13,14]. PHZ production imparts deep orange pigmentation to *P. chlororaphis* PA23 in glucose medium, while growth on octanoate results in colonies with a pale-yellow color, indicating reduced PHZ production. 

Fluorescent pseudomonads are known to produce biodegradable polyhydroxyalkanoates (PHAs) in excess carbon conditions when other nutrients such nitrogen, phosphorus, or oxygen are limiting. The PHAs are stored as granules in the cytoplasm and used as a carbon and energy source under carbon-limited conditions [15]. PHA and PHZ synthesis both appear to be important traits for root colonization and plant growth promotion in rhizosphere bacteria [16]. *P. chlororaphis* can produce medium-chain-length (mcl-) PHAs consisting of C6 to C14 carbon chain monomers when grown on vegetable oils [17,18,19,20] and fatty acids extracted from glycerol bottom [21] as sole carbon sources. In contrast, *P. putida*, which also produces mcl-PHA polymers, cannot utilize vegetable oils directly for PHA production because it does not secrete extracellular lipase [22]. The *P. chlororaphis* PA23 genome encodes a *pha* operon containing seven PHA genes, which are highly conserved among *Pseudomonas* species. 

PHA can be synthesized from related (fatty acids) or unrelated carbon substrates (glucose and gluconate). Both de novo syntheses of fatty acids and degradation of fatty acids supply precursors for PHA biosynthesis. PHZs and PHAs are secondary metabolites that accumulate during stationary phase and compete with each other for carbon partitioning or electrons or for reducing power. Production of different secondary metabolites is tightly regulated in terms of transcription and translation of specific genes as well as for partitioning of carbon. Earlier experiments have shown higher production of PHAs in *P. chlororaphis* PA23 mutants defective in PHZ production [23] and *rhlB* and *rhlR* mutants of *P. aerugenosa* defective in rhamnolipid biosynthesis [24], indicating that different secondary metabolites compete for precursors and reducing power. 

A number of mutations in regulatory gene systems, including the *gacA/gacS* two-component system, the stationary phase sigma factor *rpoS*, and the stringent response (*relA/spoT*), are known to affect biocontrol activity of *P. chlororaphis* PA23 by altering antibiotic synthesis [3,25,26,27,28]. Further positive and negative interactions among these genes regulate secondary metabolite production and fungal antagonism [25,26,27,28,29,30]. 

Scattered information is available on the effect of these mutations on PHA production in different *Pseudomonas* species. Mutations in the global regulatory system *gacA/gacS* in *P. putida* CA-3 and *P. aeruginosa* down-regulated the expression of *pha* genes, and therefore synthesis and accumulation of PHA [30]. In *P. putida* and *P. chlororaphis* 1391, the *rpoS* gene is expressed in stationary phase and positively regulates PHA synthesis after the onset of the stationary phase [26,31,32]. In *Ralstonia eutropha* H16 *spoT2* (pppGpp synthase), mutants synthesized only minor amounts of PHB [33,34]. However, in *P. putida* KT2440, there was no effect of *relA/spoT* mutations on the accumulation of PHAs [35]. 

In an earlier study examining the relationship between PHZ and PHA production, we discovered that the PHZ-minus mutant *P. chlororaphis* PA23-63 accumulated greater concentrations of mcl-PHAs compared to the wild type [23]. In the current study, we sought to determine whether changes in carbon partitioning and/or altered *pha* gene expression account for the higher PHA production observed for PHZ and PRN mutants. Further, we investigated whether regulatory genes that control PHZ/PRN also affect PHA production and transcription of *pha* genes.

## 2. Materials and Methods

### 2.1. Bacterial Strain/Mutants and Primers

*Pseudomonas chlororaphis* PA23 was isolated from the soybean rhizosphere [2]. PA23 mutants defective in PHZ and PRN production, together with primers used in the current study are listed in Table 1. The *P. chlororaphis* cultures (Wt and 6 mutants) used in the present study were described earlier for PHZs, PRN, and fungal antagonistic activity [3,12,13,25]. Mutant PA23-63 has a *Tn*5 insertion in the *phzE* gene and is not able to synthesize PHZ. Mutant PA23-8 has *prnBC* deletion and is not able to produce PRN. Mutant PA23-63-1 was derived from PA23-63 and contains a deletion of the *prnBC* genes. The other three strains have mutations in regulatory genes *gacS*, *rpoS* and SR (stringent response *relS/spoT*). *P. chlororaphis* strains PA23, PA23-8 (prn^-^), *rpoS*, and SR produced orange colonies on LB agar plates while *P. chlororaphis* strains PA23-63, PA23-63-1, and *gacS* produced white colonies on LB agar plates. Primers were designed based on the PA23 genome sequence [36] and synthesized by Alpha DNA (Montreal, QC, Canada).

### 2.2. Culture Media and Growth Conditions

Luria–Bertani (LB) medium, with 2% agar, was used to maintain *P. chlororaphis* PA23 cultures. LB agar plates containing gentamicin or rifampicin and tetracycline were streaked with *P. chlororaphis* mutants and incubated at 30 °C for 24 hours (h). Single colonies were then picked and used to inoculate liquid LB medium. Ramsay’s Minimal Medium (RMM) was used in all the experiments: 6.7 g of Na_2_HPO_4_·7H_2_O, 1.5 g of KH_2_PO_4_, 1.0 g of (NH_4_)_2_SO_4_, 0.2 g of MgSO_4_·7H_2_O, 60 mg of ferrous ammonium citrate, 10 mg of CaCl_2_·2H_2_O, 1 mL of trace element solution, pH 7.0. Each liter of trace element solution contained the following: 0.3 g of H_3_BO_3_, 0.2 g of CoCl_2_·6H_2_O, 0.1 g of ZnSO_4_·7H_2_O, 30 mg of MnCl_2_·4H_2_O, 30 mg of NaMoO_4_·2H_2_O, 20 mg of NiCl_2_·6H_2_O, 10 mg of CuSO_4_·5H_2_O. Glucose (20 g/L) or octanoic acid (20 mmol/L) was added to the RMM as the sole carbon source in the mcl-PHA production studies [23].

### 2.3. Growth of P. chlororaphis Strains and Analyses of PHA Synthesis

Single colonies of *P. chlororaphis* PA23 and derivative strains grown on LB agar plates were used to inoculate cultures with 10 mL of LB broth. Pre-experimental inoculum cultures grown in LB medium for 18 hours (h) on a rotary shaker (150 rev/min) at 30 °C were used to inoculate overnight cultures in RMM (50 mL) with 20 g/L glucose or 20 mmol/L octanoic acid as substrates, and incubated for 24 h at 30 °C. Experimental cultures containing RMM (100 mL) with glucose or octanoic acid in 500 mL baffled-flasks were inoculated with 2% (v/v) of the overnight inoculum cultures and incubated for up to 48 h on a rotary shaker (150 rev/min) at 30 °C. Cultures were harvested by centrifugation at 4190× *g* at 4 °C for 30 min.

All tests were conducted with three independently replicated cultures (i.e., 3 biological replicates). The harvested cells were washed twice in phosphate-buffered saline and dried at 60 °C for 48 h to estimate cell dry mass. PHA subunit composition was determined by gas chromatography (Agilent Technologies Canada Inc., Mississauga, ON, Canada, Model 7890A) analysis, as described by Braunegg et al. [37]. Samples of dried cell biomass (5–10 mg) were placed in 15 mL screw-cap tubes and analyzed for percent cell dry mass (% cdm) accumulation of PHA and subunit composition. To this biomass, 1 mL of chloroform containing 1 mg of benzoic acid [internal standard], and 1 mL of methanol containing 15% concentrated sulfuric acid were added. Tubes were boiled in a water bath for 6 h, and then 0.5 mL of water was added to each tube. The chloroform (lower) layer was transferred to a 2 mL GC vial and analyzed by GC fitted with a DB-23 capillary column and flame ionization detector. The initial oven temperature was maintained at 60 °C for 5 min, which was increased to 250 °C at a ramping rate of 15 °C/min. The peaks were identified by their retention time, and concentration of subunits was estimated by comparison with known concentrations of different 3-hydroxy fatty acid standards.

### 2.4. PHA Production by P. chlororaphis PA23 at Different Time Points 

Cultures of *P. chlororaphis* PA23 and derivative strains were prepared as described above (Section 2.3). The experimental cultures were incubated for up to 96 h on a rotary shaker [150 rev/min] at 30 °C. Samples for cell dry mass and PHA production were analyzed at *t* = 12, 24, 36, 48, 72, and 96 h post-inoculation (h pi), as described earlier. After harvesting the cells, supernatants were used for estimating residual glucose, octanoic acid and ammonium nitrogen in the medium [23]. For glucose, estimation, supernatants (1 mL) were centrifuged at 21,000× *g* for 10 min and glucose was estimated using High Performance Liquid Chromatography (Water Breeze HPLC; System, Water Incorporation, Milford, MA, USA). For octanoic acid, supernatants were vortexed for 3 min and 1 mL of supernatant in a 15-mL culture tube was dried in an oven for 48 h at 60 °C. The residues were methanolyzed and octanoic acid concentrations were determined by GC analysis, described above (Section 2.3). Ammonium nitrogen was measured using the Quikchem method 10-107-06-1-I for determination of ammonium in wastewater by flow injection analysis (Lachat Instrument, Loveland, CO, USA). 

### 2.5. RNA Isolation, cDNA Synthesis and Gene Expression 

To monitor the expression of metabolite and regulatory genes involved in PHZ and PHA production, quantitative real-time PCR (qPCR) was employed. The expression of *phaC1*, *phaZ*, *phaC2*, *phaD*, *phaF* and *phaI* (all associated with PHA production) was determined in *P. chlororaphis* PA23 and its derivative strains. The housekeeping gene, *rpoB*, was chosen to normalize expression values of the target genes. Expression of these genes in RMM glucose medium (supporting PHZ production) or RMM octanoic acid medium (supporting PHA production) was also compared. Relative gene expression in comparison to Wt *P. chlororaphis* PA23 was calculated. Primers used for gene expression analysis are listed in Table 1. Primers were designed based on the sequences of the respective genes obtained from *P. chlororaphis* PA23 (GenBank accession no. NZ_CP008696). 

Cultures were grown in RMM (2% glucose) or RMM octanoic (20 mmol/L) at 30 °C for 24 h or 20 h, respectively. A sample (0.2 mL) for each time point was mixed with 0.2 mL RNA protect, vortexed, and then stored at −80 °C until required for RNA isolation. Total RNA was extracted from the cell pellets using a PureLink RNA Mini Kit (Ambion, Life Technologies, Carlsbad, CA, USA) following the supplier’s protocol. Residual DNA was removed through on column DNase treatment using the Pure link DNase system (Thermo Fisher Scientific, Waltham, MA, USA). The concentration of RNA was determined using a Nanodrop 1000 spectrophotometer (Thermo fisher Scientific, Waltham, MA, USA), and RNA integrity was analyzed by electrophoresis using an Experion system (Bio-Rad Laboratories Canada, Mississauga, ON, Canada). cDNA was generated from 1 μg RNA by reverse transcription using the Maxima First-Strand cDNA Synthesis Kit (Thermo Scientific, Waltham, MA, USA) and the following conditions were employed: initial heating at 25 °C for 10 min, reverse transcription at 50 °C for 15 min, and enzyme denaturation at 85 °C for 5 min. 

Gene expression was studied by qPCR using the CFX96 C1000 Touch Thermocycler Real-Time System (Bio-Rad Laboratories Canada, Mississauga, ON, Canada) and SsoFast SYBR Green Supermix (CFX96 C1000 Touch Thermocycler Real-Time System (Bio-Rad Laboratories Canada, Mississauga, ON, Canada)) using Hard Shell PCR 96-well thin walled plates (CFX96 C1000 Touch Thermocycler Real-Time System (Bio-Rad Laboratories Canada, Mississauga, ON, Canada)). PCR reactions were performed in a 10-μL reaction volume containing 5 μL SsoFast SYBR Green Supermix, 0.5 μL each of forward and reverse primer, 1 μL cDNA, and 3 μL of RNAse/DNAse free PCR water. PCR reaction conditions included an initial denaturation at 98 °C for 3 min, followed by 40 cycles of 98 °C for 10 seconds (s) and 60 °C for 30 s. The reactions were performed in triplicate and experiments were repeated with three biological replicates. Relative gene expression was determined using the ΔΔCt method [38]. The data was analyzed using Cfx96 Manager software version 3.1 (Bio-Rad Laboratories Canada, Mississauga, ON, Canada).

### 2.6. Statistical Analysis

Cell dry mass and PHA production data was analyzed by two-way analysis of variance (ANOVA) using SAS analytic software (SAS Institute Incorporation, Cary, NC, USA (https://www.sas.com). Least significant differences (LSD) for interactions between substrate and treatments were calculated and are shown on the histograms.

## 3. Results

### 3.1. pha Cluster in P. chlororaphis PA23

Expression of six *pha* genes of *P. chlororaphis* PA23 encoded by EY04_01515, EY04_01520, EY04_01525, EY04_01530, EY04_01535, and EY04_01540 was studied in the present investigation. Two Class II PHA synthase genes, *phaC1* and *phaC2*, are encoded in the *P. chlororaphis* PA23 genome. A PHA depolymerase gene, *phaZ*, is located in between *phaC1* and *phaC2*. A putative polyhydroxyalkanoic acid protein (EY04_01510), identified as a hypothetical protein, resides in the *P. chlororaphis* PA23 *pha* operon together with three regulatory genes, *phaD*, *phaF*, and *phaI*. The nucleotide sequence of these genes from the whole genome sequence from NCBI (Accession No. CP008696) was used to design primers for qPCR to study gene expression. In *P. putida* the *phaC1*, *phaZ* and *phaC2*, *phaF* and *phaI* genes have their own promoter, which is regulated by regulated by *phaD* as well as *phaFI* products [39]. The *phaF* and *phaI* genes are transcribed divergently to other *pha* genes [39]. The two *phaC1* and *phaC2* gene products encoding PHA synthases have different substrate specificity [40]. 

### 3.2. Growth and Cell Dry Mass of P. chlororaphis 23 and It Mutant Derivatives

**Figure 1:**
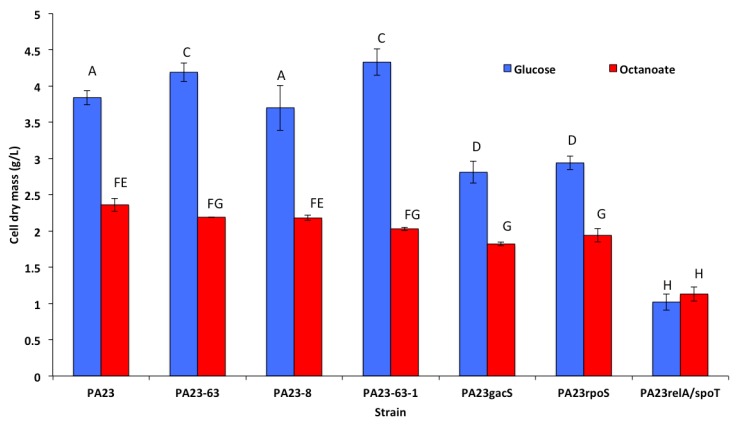
Cell dry mass production by *P. chlororaphis* PA23 and it mutant derivatives in RMM medium with glucose versus octanoic acid as the carbon source. Bars with same letter are non-significantly different at the 5% level.

*P. chlororaphis* PA23 and its mutant derivatives were able to grow in RMM with glucose and octanoate as a sole carbon source. The overall growth of bacteria was better in RMM with glucose compared to RMM with octanoic acid. In glucose medium, *P. chlororaphis* PA23 accumulated 3.86 g/L cell dry mass, while in octanoic acid medium, it accumulated only 2.36 g/L cell dry mass, which was significantly different (Figure 1). Mutants defective in PHZ production produced more cell dry mass than the other mutants on glucose medium. There was a non-significant difference in cell dry mass of PHZ and PRN mutants in octonaic acid as a substrate. Cell dry mass produced by the *P. chlororaphis gacS*, *rpoS*, and SR mutants was significantly less than wild type *P. chlororaphis* PA23 in both RMM glucose and RMM octanoic acid media. 

### 3.3. PHA Production by P. chlororaphis PA23 and It Mutant Derivatives

**Figure 2:**
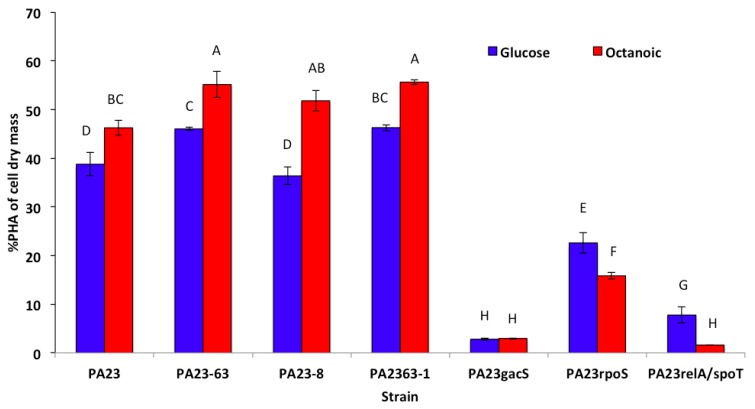
PHA accumulation by Wt *P. chlororaphis* PA23 and its mutant derivatives in RMM media with glucose versus octanoic acid as the carbon source. Bars with same letter are non-significantly different at the 5% level.

PHA production was studied in RMM media containing glucose or octanoic acid as a sole carbon source. Among the three mutants defective in PHZ and PRN synthesis, only two mutants *P. chlororaphis* PA23-63 and *P. chlororaphis* PA23-63-1 produced significantly greater concentrations of mcl-PHAs than Wt *P. chlororaphis* PA23 (Figure 2). In the case of mutants defective in PRN synthesis, there was no significant increase in PHA accumulation in *P. chlororaphis* PA23. PHA accumulation in RMM octanoic acid medium was also affected significantly in the PHZ and PRN mutants. PHA accumulation increased by 19.2%, 11.97%, and 20.28% over the Wt in *P. chlororaphis* PA23-63, PA23-63-1, and PA23-8 respectively. In the *gacS* mutant, PHA accumulation was only 2.81% and 2.97% of cell dry mass as compared to 34.16% and 46.25% for the Wt in RMM glucose and RMM octanoate media, respectively. PHA accumulation in the *P. chlororaphis rpoS* mutant decreased significantly to 22.59% and 15.83% in RMM glucose and RMM octanoic acid media, respectively. In the *P. chlororaphis* SR mutant, PHA accumulation was only 7.78% and 1.62% in RMM glucose and octanoic acid medium, respectively, which was significantly less than Wt *P. chlororaphis* PA23.

### 3.4. Monomer Composition of PHAs Produced by P. chlororaphis PA23 and It Derivative Mutants

**Figure 3:**
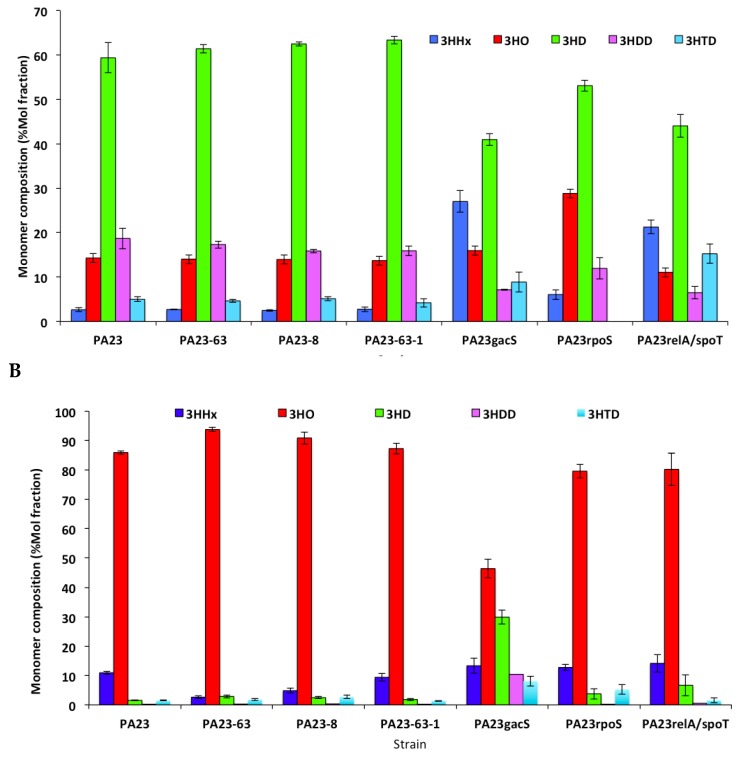
Monomer composition of PHAs synthesized by *P. chlororaphis* PA23 and its mutant derivatives in (**A**) RMM glucose medium and (**B**) RMM octanoate medium as the carbon source.

In RMM medium with glucose, *P. chlororaphis* PA23 synthesized PHAs with 3-hydroxydecanoate as a major component (app. 60 mol%; Figure 3). The other monomers included 3-hydroxyhexanoate (2.6 mol%), 3-hydroxyoctanoate (14.2 mol%), 3-hydroxydodecanoate (18.7 mol%), and 3-hydroxytetradecanoate (5.0 mol%). The PHZ and PRN defective mutants synthesized PHA polymers with subunit compositions similar to those of the Wt. For the three regulatory mutants (*gacS*, *rpoS*, SR), the PHAs contained higher amounts of 3-hydroxyhexanoate and/or 3-hydroxyoctanoate compared to the parent. The monomer composition of PHAs synthesized by *P. chlororaphis* PA23 in RMM octanoate medium was different from that of RMM glucose medium.

The major subunit components of PHAs synthesized in RMM octanoate medium were 3-hydroxyoctanoate (86 mol%), followed by 3-hydroxyhexanoate (11 mol%). Other monomers like 3-hydroxydecanoate, 3-hydroxydodecanoate, and 3-hydroxytetradecanoate were also present in small quantities. PHAs produced by PHZ and PRN mutants had greater 3-hydroxoctanoate content and lower 3-hydroxyhexanoate content than the Wt *P. chlororaphis* PA23 strain. In this carbon source, the *gacS* mutant showed a different monomer composition of all five PHA monomers compared to PA23. 

### 3.5. Growth and PHA Production by P. chlororaphis PA23 in Glucose and Octanoic Acid Medium at Different Time Points

**Figure 4:**
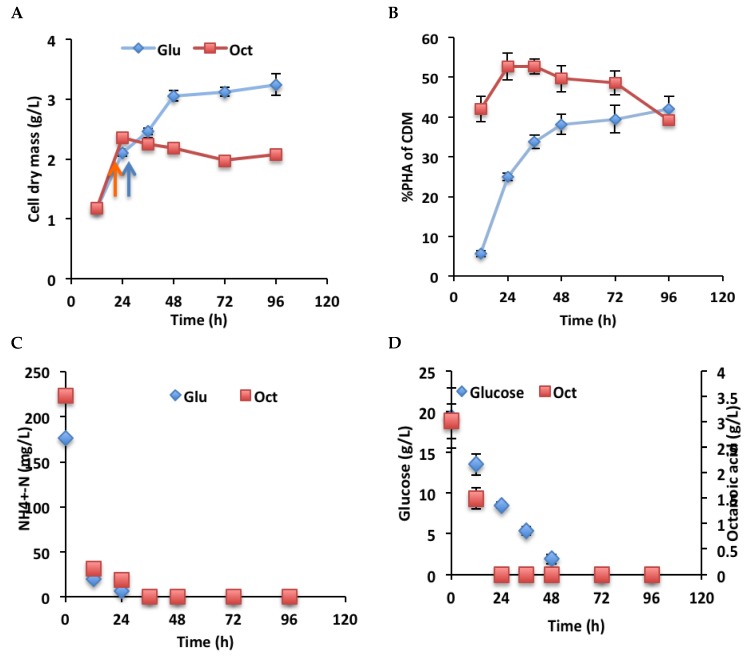
Growth and PHA accumulation, with substrate and nitrogen consumption by *P. chlororaphis* PA23 in RMM glucose versus RMM octanoic acid media. (**A**) Cell dry mass production; (**B**) PHA accumulation; (**C**) Residual glucose and octanoic acid concentrations; and (**D**) Residual ammonium-nitrogen concentrations. Samples for RNA isolation from RMM glucose cultures were taken at 24 h pi and from RMM octanoic acid cultures at 16 h pi.

*P. chlororaphis* PA23 was grown in RMM glucose or RMM octanoic acid media for 96 h and samples were analyzed for cell dry mass, PHA accumulation, residual ammonium-N, and residual glucose or octanoic acid (Figure 4). Cell dry mass in RMM glucose medium increased up to 48 h pi and then stabilized with no further increase in cell dry mass. In RMM octanoic acid medium, cell dry mass increased up to 24 h pi, with no further increase in cell dry mass thereafter. PHA production in RMM glucose medium increased up to 96 h pi, but in RMM octanoic acid medium, maximum PHA accumulated at 24 h pi with no further increase up to 72 h pi. In RMM glucose and RMM octanoic media, maximum ammonium-N was consumed within 24 h pi and no detectable ammonium nitrogen was present in the medium after 36 h pi. Samples for qPCR were taken at 24 h pi in RMM glucose medium and at 16 h pi in RMM octanoic acid medium, respectively. At these time points, both cultures had started to accumulate PHA polymers (Figure 4B).

### 3.6. Expression of the phzA Gene in Mutants Defective in PHZ and/or PRN Production 

**Figure 5:**
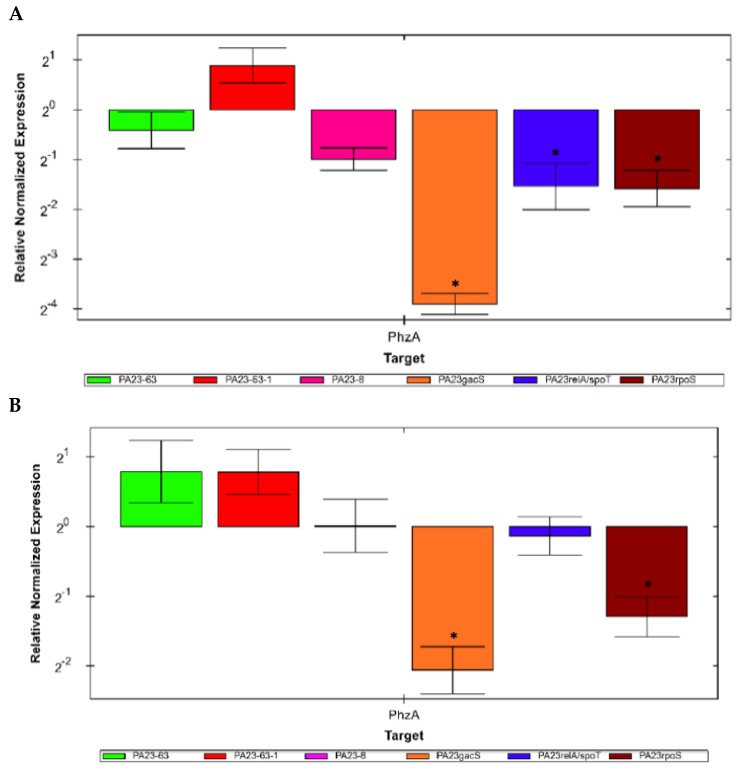
Expression of *phzA* in *P. chlororaphis* PA23 mutants defective in the PHZ and/or PRN structural genes or in genes that regulate PHZ and PRN synthesis in cultures containing Ramsay’s Minimal Medium (RMM) plus (**A**) glucose or (**B**) octanoic acid as the carbon source. The housekeeping gene *rpoB* was chosen to normalize expression values of target genes. Expression levels in the Wt were normalized to 1. Data was normalized and expression relative to wild type PA23 was calculated. Differentially expressed genes are indicated with an asterisk (* *p* < 0.01).

The first gene in the PHZ biosynthetic operon is *phzA* (EY04_26270, annotated as PHZ biosynthesis protein). Expression of *phzA* was studied by qPCR in *P. chlororaphis* PA23 (Wt), PA23-63 (*phz^-^*), PA23-8 (*prn^-^*), and PA23-63-1 (*phz^-^ prn^-^*), as well as the *gacS*, *rpoS*, and SR mutants. There was no change in *phzA* gene expression in the *phz^-^*, *prn^-^*, or *phz^-^ prn^-^ P. chlororaphis* mutants in either RMM glucose or RMM octanoic acid cultures (Figure 5). In the *gacS*, *rpoS*, and *relA/spoT* regulatory mutants, there was a significant decrease in the expression of *phzA* in all three strains grown in glucose; in octanoate, *phzA* expression was reduced in PA23gacS and PA23rpoS. 

### 3.7. Expression of pha Genes in the PA23 PHZ and/or PRN Mutants

**Figure 6:**
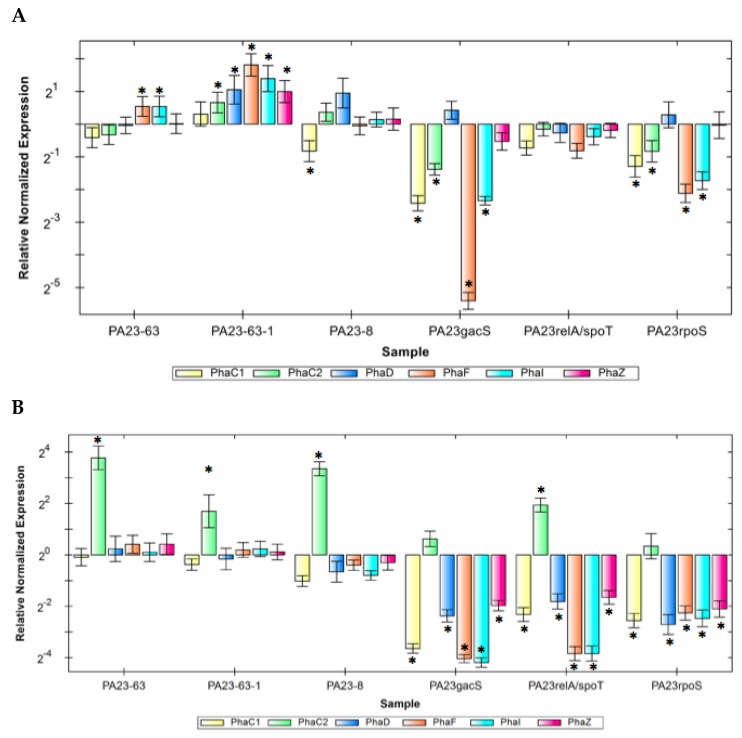
Expression of *pha* genes in *P. chlororaphis* PA23 mutants defective in the PHZ and/or PRN structural genes or in genes that regulate PHZ and PRN synthesis in cultures containing Ramsay’s Minimal Medium (RMM) plus (**A**) glucose or (**B**) octanoic acid as the carbon source. The housekeeping gene *rpoB* was chosen to normalize expression values of target genes. Expression levels in the WT were normalized to 1. Data was normalized and expression relative to wild type PA23 was calculated. Differentially expressed genes are indicated with an asterisk (* *p* < 0.01).

Six *pha* genes, *phaC1, phaZ, phaC2, phaD, phaF*, and *phaI* were studied in *P. chlororaphis* PA23, PA23-63 (*phz*^-^), PA23-8 (*prn*^-^), and PA23-63-1 (*phz^-^ prn*^-^). In PA23-63 (*phz*^-^) grown in RMM glucose, no change in expression levels of *phaC1*, *phaZ, phaC2*, and *phaD* were observed (Figure 6). However, two *pha* genes, *phaF* and *phaI*, were up-regulated significantly. In PA23-8 (*prn*^-^), no change in the *pha* genes was observed, except for the *phaC1* gene, which was down-regulated significantly. In PA23-63-1 (*phz^-^ prn*^-^), expression levels of *phaZ*, *phaD, phaF*, and *phaI* genes were significantly up-regulated. In RMM octanoic acid, expression levels of the *phaC2* gene were significantly increased in PA23-8 (*prn*^-^) and the double mutant PA23-63-1 (*phz^-^ prn*^-^). 

**Figure 7:**
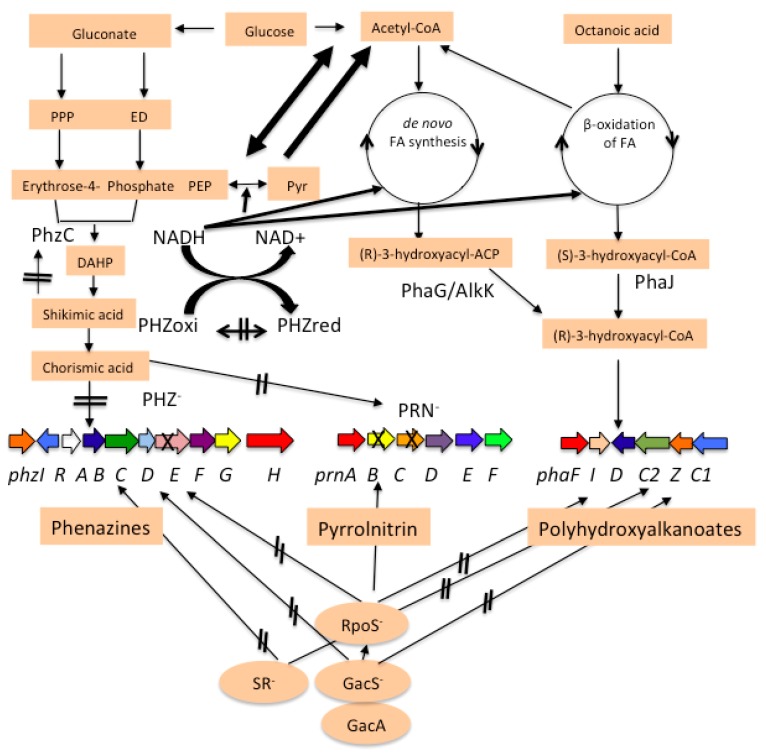
Pathway of PHZ/ PRN and PHA production. Blocking PHZ and PRN production provides more precursors for PHA production. X indicates the gene mutated or deleted in *phz* and *prn* gene clusters. Thick arrows indicate higher carbon and reducing power flow for PHA production.

Three regulatory mutants PA23gacS, PA23rpoS, and SR (*PA23relA/spoT*) affecting PHZ and PRN production were studied for expression of *pha* genes in glucose and octanoic medium (Figure 7). When grown in RMM glucose, the *gacS* and *rpoS* mutants both exhibited reduced expression of *phaC1*, *phaC2*, *phaF*, and *phaI* genes, while *phaZ* and *phaD* remained unaffected. In the *P. chlororaphis* SR (PA23*relA/spoT*) mutant, there was no significant change in the expression of any of the *pha* genes. By way of comparison, expression of *phaC1*, *phaD*, *phaF*, *phaI*, and *phaZ* was down-regulated in *P. chlororaphis* PA23gacS, *PA23*rpoS and the SR (PA23*relA/spoT*) mutants grown in octanoic acid medium. Surprisingly, *phaC2* was significantly upregulated in the SR mutant. 

## 4. Discussion

Fluorescent pseudomonads are known to produce more than 100 phenazine-like compounds, which have been classified as antibiotics. Phenazines have a role in the survival of bacteria under low oxygen tension, iron acquisition, redox homeostatis, and transition to biofilm growth, which give producers an ecological advantage in the environment [6]. *P. chlororaphis* PA23 was identified as a biocontrol bacterium due to the production of PHZ and PRN antibiotics [4]. This bacterium was also able to use vegetable oils as a sole carbon source to produce mcl-PHAs [23]. PHA is an energy and carbon storage polymer, which acts as food reserve for bacteria. The polymer is degraded under starving condition when nitrogen is present in the environment. Presence of PHAs in rhizosphere bacteria enhances their colonization and promotes plant growth. PHA producing bacteria can survive under starving conditions and under hostile environments. Therefore, both PHZ/PRN and PHA provide ecological benefits to the producing bacteria.

A number of mutants defective in the production of PHZ and PRN antibiotics are available [12,13,14,25]. Our preliminary studies indicated that mutants of *P. chlororaphis* PA23 defective in PHZ production accumulated more PHA than the PA23 parent strain. Both PHZ/PRN and PHA are accumulated in the stationary phase of *P. chlororaphis* growth and have common global regulators like GacA/GacS, RpoS, and the stringent response (SR). The precursors for PHZ and PRN production are acetyl-CoA and erythrose-4-phosphate, which are provided by β-oxidation and by the ED pathway, respectively [41] (Figure 7). 

Glucose is a preferred carbon source for cell growth and PHZ synthesis, but not for PHA production. *P. chlororaphis* PA23 produced a deep orange color and 38.5% more cell dry mass in glucose medium with 34% PHAs. In octanoic medium, it produced pale yellow color with low cell dry mass but with higher PHAs (46.25%). Moreover, PHA accumulation was higher in *P. chlororaphis phz*^-^, *prn*^-^, and *phz*^-^ *prn*^-^ mutants cultured on RMM octanoate medium than RMM glucose medium. Higher PHA production in cultures containing fatty acids as the carbon source is correlated with higher gene expression levels of *phaC1*, *phaC2*, and *phaZ*, which was at least 15-fold greater in cells grown on fatty acid than those in cells grown on glucose in *P. putida* KT2440 [42]. Whether the enhanced PHA production was due to a higher expression of *pha* genes in PHZ/PRN null mutants or change in metabolic flux for PHA production by supplying more carbon expression of pha genes was studied.

To explore the first possibility, we studied the expression of *pha* genes in *P. chlororaphis phz*^-^, *prn*^-^, and *phz*^-^ *prn*^-^ mutants (PHZ/PRN null mutants). No significant changes in the levels of *phaC1* and *phaC2* gene transcription were observed in *P. chlororaphis phz*^-^, *prn*^-^, and *phz*^-^ *prn*^-^ mutants, compared with the expression levels of these genes in *P. chlororaphis* PA23 cultured in RMM glucose medium. However, when the *P. chlororaphis phz*^-^, *prn*^-^, and *phz*^-^ *prn*^-^ mutants were cultured in RMM octanoic acid medium, expression levels of the *phaC2* gene increased significantly [4–8 times] compared to *P. chlororaphis* PA23 cultured in the same medium. The greater level of PHA production and accumulation in the *P. chlororaphis phz*^-^, *prn*^-^ and *phz*^-^ *prn*^-^ mutants cultured in RMM octanoic acid could be due to higher *phaC2* expression levels. Earlier, the expression of *phaC2* along with *phaC1* was associated with greater PHA production and accumulation than the *phaC1* gene alone in *Pseudomonas corrugata* [43]. Moreover, PhaC1 and PhaC2 have different substrate specificity to use different carbon sources efficiently [40].

Common global regulators like GacS/GacA, RpoS and SR are involved in PHZ/PRN and PHA production. The GacA/GacS and RpoS either abolished or decreased the PHZ/PRN and PHA production, while the SR system had little or no effect. These systems also regulated the transcription of *phz*, *prn* and *pha* genes [30]. The transcription of *phaC1*, *phaZ*, *phaD*, *phaF* and *phaI* was significantly downregulated in octanoate medium in *gacA/gacS* and *rpoS*. SR mutants (*relA/spoT*) of *P. chlororaphis* PA23 were impaired in PHA production with an effect on transcription of *pha* genes in glucose medium. In this respect, *P. chlororaphis* PA23 behaved like *Ralstonia eutropha* in PHA production [33,34]. The reduced *phzA* transcriptional fusion expression and decreased PHZ production in *P. chlororaphis* was identical to *pha* gene expression and decreased PHA production [12]. Regulation of PHZ/PRN and PHA synthesis by the GacA/GacS, RpoS and SR systems suggests that the expression of the *phzA* and *pha* genes is under the influence of global regulatory networks, but limited information is available regarding how these regulatory pathways are interlinked. 

Blocking of one secondary metabolite can enhance the production of other secondary metabolites. PHZ and PRN in *P. chlororaphis* are synthesized via the shikimic pathway and precursors are provided by the ED and fatty acid biosynthesis/degradation pathways [44]. *phz^-^/prn^-^* mutants of *P. chlororaphis* are blocked to produce PHZ/PRN and shikimic acid and chorsimic acid accumulated in the medium, Shikimic acid and chorismic acid, which inhibited their production by feedback and allosteric effects [45]. Blocking of aromatic amino acids synthesis by deleting shikimic acid kinases was earlier reported to accumulate shikimic acid in the medium [46] and the excess carbon can be diverted for other secondary metabolites like PHAs. In the *phz^-^* mutant *P. chlororaphis* PA23-63, higher levels of PRN production are observed compared to Wt PA23. The defect in PHZ production resulted in greater carbon flow (C3 and C4 organic phosphates) into the shikimic acid pathway for PRN synthesis, which led to increased PRN production [12]. Blocking of metabolic pathway for pyocyanin, pyochelin, anthranilate and prephenate production in *P. aeruginosa* PA1201 increased PHZ production by 46%, 14%, 15%, and 23% respectively [47]. The *phzC* gene encodes a type II DAHP synthase in PHZ-producing bacteria. This step is tightly regulated by regulated feedback inhibition; otherwise, it will deplete the metabolites for amino acid biosynthesis. Therefore, it is possible that a mutation in the *phz* and *prn* genes, which resulted in defective PHZ and PRN proteins, shunted more carbon to the PHA synthesis pathway, resulting in greater PHA accumulation. Phenazine production in *P. fluorescens* 2–79 was reported as high as 0.31 g/g cell biomass, which is comparable to 0.25–0.4 g/g of cell biomass PHA production [48]. 

Metabolic engineering has been used to increase PHZ or PHA production in *Pseudomonas* species. Blocking of metabolic pathways, which diverts precursors to other futile pathways, overexpressing the genes of feeding pathways or deleting regulatory genes are common strategies to enhance PHZ production in *P. aeruginosa* and *P. chlororaphis* [49,50]. Deletion of the *prn* operon showed that PHZ production increased 2.5 times in *P. chlororaphis*, which could be due to the higher availability of precursors for PHZ production [12]. Phenazines and PRNs are redox active compounds modulating the intracellular redox potential and serving as intracellular redox buffers [51]. PHZ^-^ mutants of *P. aeruginosa* PA14 have higher NADH/NAD^+^ redox ratio than wild type PHZ producing cultures, which was correlated with oxygen limitation. The ratio of NADH/NAD^+^ plays a major role in central metabolism [44]. Oxygen limitation has been earlier reported to improve PHA production in *P. putida* [52]. These conditions are more suitable for PHA accumulation in PHZ^-^ mutants. In *P. putida* acetyl-CoA/free CoA and NADH/NAD^+^ ratios regulate PHA storage/mobilization [53]. Phenazine reduction was at the peak during the stationary phase when PHA production started. Higher reducing power in PHZ^-^ mutants could lead to higher PHA production in *P. chlororaphis*. Overproducing PHA strains of *P. putida* have low NADPH/NADP^+^ ratios because more NADPH and NADH are consumed to convert 3-ketoacyl-ACP into (R)-3-hydroxyacyl-ACP [54]. High NADH/NAD+ ratio supports high PHA production. PHAs act not only as carbon and energy reservoirs, but also as a sink for reducing power. Similar observations of the importance of the NADH/NAD+ ratio for PHA synthesis from fatty acids in *P. putida* have been published [55,56,57]. Recently PHZ-producing recombinants of *P. putida* KT2440 have been developed. These recombinants produced up to 424 mg/L PHZ. However, PHA production in these recombinants was not studied [58]. It will be interesting to see how PHA production was affected in these recombinants or how the deletion of *pha* genes in these recombinants affects PHZ production.

## 5. Conclusions

*Pseudomonas chlororaphis* PA23 produces a number of compounds, including phenazines (PHZ), pyrrolnitrin (PRN), hydrogen cyanide (HCN), proteases, lipases and siderophores, which contribute to its biological control potential. It can also use vegetable oils to accumulate medium chain length polyhydroxyalkanoates (PHAs). Phenazine/pyrrolnitrin defective mutants produced more PHAs from glucose and octanoic acid than *P. chlororaphis* PA23. Both PHA and PHZs are accumulated during stationary phase and have common regulators like GacA/GacS, RpoS, and the stringent response, which affect their expression. The possible reason for enhanced PHA production and expression of *pha* genes was studied in these mutants. Mutants defective in PHZ and PRN production showed an increase in expression of *pha* genes. However, regulatory mutants i.e., *gacS*, *rpoS* and SR mutants had significant effect on *pha* gene expression and PHA production. It was speculated that increased PHA production in these mutants could be due to availability of more carbon and reducing power in PHZ and PRN mutants. How PHZ/PRN and PHA production pathways are connected is not very clear. Recombinants of *P. putida* producing PHZ/PRN will throw some light on production of these two important secondary metabolites. 

## Figures and Tables

**Table 1:** Bacterial strains and oligonucleotide primers used in the present study.

Strain/Mutant	Relevant Genotype, Phenotype or Sequence	Reference
*P. chlororaphis* PA23	Rif^R^; WT [soybean root tip isolate] PHZ^+^PRN^+^	[2]
PA23-63	PHZ^-^ Rif^R^ *phzE*::Tn5-OT182 genomic fusion	[12]
PA23-8	PRN*^-^* Rif^R^ *prnBC* deletion mutant	[12]
PA23-63-1	PHZ^-^ PRN^-^ Rif^R^ *phzE*::Tn5-OT182 genomic fusion; *prnBC* deletion mutant	[12]
PA23rpoS	PA23 with pKNOCK inserted into *rpoS*	[25]
PA23gacS	PHZ^-^ Rif^R^ *gacS*::Tn5-OT182 genomic fusion	[3]
PA23relA/spoT	PA23 with pKNOCK-Gm inserted into *relA*; Tet^R^ cassette inserted into *spoT*	[25]
PA23rpoS	PA23 with pKNOCK-Tc inserted into *rpoS*	[13]
**Primers**		
*phzA*-FOR	5′-GACTGGCAATGGCACAAC-3′	[14]
*phzA*-REV	5′-GCAATAACCTTCGGGATAACC-3′	[14]
*phzI*-FOR	5′-CGATGCCGTTGTTCTGG-3′	[14]
*phzI*-REV	5′-AGCCGTTCGTAGTGGACTC-3′	[14]
*phaF*-FOR	5′-GAAAAAGAAGGCAGCTCGTG-3′	This study
*phaF-*REV	5′-ATCGACTTTCTTGCCGACAG-3′	This study
*phaI*-FOR	5′-CTACACCAAGGTCGGTCAGG-3′	This study
*phaI*-REV	5′-ATCCAGCTGCACTTCGACTT-3′	This study
*phaD*-FOR	5′-CTGGGTATCGCTGACCAGTT-3′	This study
*phaD*-REV	5′-ACTACCGCTTCCTGTTCCAG-3′	This study
*phaC2*-FOR	5′-ATTCCAGATCAGGTCGTTGG-3′	This study
*phaC2*-REV	5′-GGTCAGCCTGCTGGATAGTC-3′	This study
*phaZ*-FOR	5′-CCTGCCCATAGTCGAGGTAA-3′	This study
*phaZ*-REV	5′-CTGGAGCTGGTGTTTCCATT-3′	This study
*phaC1*-FOR	5′-GCCAGGTAGGTTTGCAGGTA-3′	This study
*phaC1-*REV	5′-TCTGCTCGTATGGTGCTGAC-3′	This study
